# Prodromal Symptoms in Patients Presenting With Myocardial Infarction

**DOI:** 10.7759/cureus.43732

**Published:** 2023-08-18

**Authors:** Hina Sohail, Jaghat Ram, Amjad A Hulio, Sajid Ali, Muhammad N Khan, Najia A Soomro, Muhammad Asif, Sidrah Agha, Tahir Saghir, Jawaid A Sial

**Affiliations:** 1 Interventional Cardiology, National Institute of Cardiovascular Diseases, Karachi, PAK; 2 Interventional Cardiology, National Institute of Cardiovascular Diseases, Larkana, PAK; 3 Electrophysiology, National Institute of Cardiovascular Diseases, Karachi, PAK; 4 Cardiology, Liaquat National Hospital, Karachi, PAK; 5 Adult Cardiology, National Institute of Cardiovascular Diseases, Karachi, PAK; 6 Cardiology, National Institute of Cardiovascular Diseases, Karachi, PAK

**Keywords:** st-segment elevation myocardial infarction, impending myocardial infarction, early warning signs, prodromal symptom, acute myocardial infarction

## Abstract

Background

Prodromal symptoms are warning signs of an impending acute myocardial infarction (AMI). However, they are often overlooked by both patients and primary clinicians, and little is known about them. Therefore, this study aims to assess the frequency and types of prodromal symptoms in patients with AMI.

Methodology

This descriptive cross-sectional study was conducted at a tertiary care cardiac center. Consecutive patients diagnosed with AMI within the last week were evaluated for prodromal symptoms. The prodromal symptoms included chest pain, chest heaviness, chest burning, palpitations, fatigue, sleep disturbance, shortness of breath (SOB), dizziness, anxiety, sudden heat or cold, back pain, and vomiting.

Results

In a sample of 242 patients, 79.6% were males, with a mean age of 54.7 ± 12.2 years, and 179 (74%) were diagnosed with ST-segment elevation myocardial infarction (STEMI). Among the participants, 142 (58.7%) showed no prodromal symptoms. Among those with prodromal symptoms, chest pain was the predominantly reported prodromal symptom with a frequency of 68%, followed by chest heaviness at 44%, palpitations at 42%, shortness of breath at 34%, and chest burning at 27%. Unusual fatigue in 23% and sleep disturbance in 22% of the patients were also reported.

Conclusion

The findings from this study revealed that prodromal symptoms were present in a significant proportion of acute myocardial infarction (MI) cases, with more than four in 10 patients reporting these early warning signs. The most commonly observed prodromal symptoms were chest pain, chest heaviness, palpitations, shortness of breath, and chest burning. The timely identification of these symptoms can help prevent infarction, thereby reducing the burden of heart failure and other related mortalities.

## Introduction

Cardiovascular diseases (CVD) are the leading cause of mortality not only in Pakistan but also worldwide. According to the World Health Organization (WHO), in 2019, 8.9 million deaths were reported due to cardiovascular diseases [1]. Prodromal symptoms usually serve as early warning signs of an impending myocardial infarction (MI). They indicate early stages of heart disease and are shorter in duration, less severe, transient, and nonspecific as compared to unstable angina, which is a prolonged, severe, and specific type of chest pain or discomfort occurring at rest; may be new onset; or occurs with less exertion than previously experienced with stable angina that may not subside with medications or rest [2]. These symptoms lack specific characteristics, are transient, and may occur days to several weeks before an adverse cardiac event. Identifying, screening, and treating patients at risk of myocardial infarction and sudden cardiac death present challenges. Many patients may die before reaching the hospital, and failure to recognize prodromal symptoms may result in increased cardiac-related morbidity and mortality [3].

The objective of this study is to assess the frequency and types of prodromal symptoms in patients presenting with acute myocardial infarction (AMI). Little is known about prodromal symptoms at the primary care level. It has been reported that many people tend to ignore these symptoms and fail to seek medical assistance. Even when they do, their clinicians may misdiagnose the condition. Nevertheless, prevention is always better than cure, and early intervention to prevent infarction can reduce the burden of heart failure and other related mortalities.

## Materials and methods

This cross-sectional study was conducted in the medical ward of the National Institute of Cardiovascular Diseases (NICVD), Larkana, over a period of six months from September 2020 to February 2021. The patients were selected using a consecutive sampling technique. The study included patients of both genders, aged ≥18 years, who had experienced ST-segment elevation myocardial infarction (STEMI) and non-ST-segment elevation myocardial infarction (NSTEMI) within the last week. Patients with a prior history of cardiac-related surgery or intervention, stable angina, or fibrinolytic treatment were excluded from the study. Additionally, patients who refused to give consent were also excluded, in accordance with the International Council for Harmonisation-Integrated Addendum to Good Clinical Practice (ICH-GCP) guidelines [4]. The study was conducted with the approval of the ethical review committee of the NICVD, Karachi (ERC-09/2020).

The baseline characteristics of the patients, along with demographic details and medical history, were recorded. In addition, prodromal symptoms such as chest pain, chest heaviness, chest burning, palpitations, fatigue, sleep disturbances, shortness of breath (SOB), dizziness, anxiety, sudden heat and cold sensations, back pain, and vomiting were assessed through interviews within two months of acute myocardial infarction.

Percentages and frequencies were calculated for categorical variables, while the mean and standard deviation (SD) were presented for numerical variables. The chi-square test was applied to determine the association of the diagnosis, gender, and age with prodromal symptoms. A P-value of <0.05 was considered statistically significant.

## Results

Among the 242 individuals who participated in the study, the majority (71.9%) were males, with a mean age of 54.7 ± 12.2 years. The demographics and baseline characteristics of the study population are shown in Table 1.

**Table 1:** Distribution of clinical data for the overall sample and stratified by the occurrence of prodromal symptoms A P-value of <0.05 was considered statistically significant. Data presented as N (%)

	Total (N)	Prodromal symptoms		P-value
		Yes	No	
Total (N)	242	100 (41.3%)	142 (58.7%)	-
Gender				
Male	174 (71.9%)	64 (64%)	110 (77.5%)	0.014
Female	68 (28.1%)	36 (36%)	32 (22.5%)	
Age				
<40 years	21 (8.7%)	10 (10%)	11 (7.7%)	0.446
41-60 years	157 (64.9%)	59 (59%)	98 (69%)	
>60 years	64 (26.4%)	31 (31%)	33 (23.2%)	
Married	239 (98.8%)	99 (99%)	140 (98.6%)	0.425
Employed	110 (45.5%)	37 (37%)	73 (51.4%)	0.009
Diagnose				
ST-segment elevation myocardial infarction	179 (74%)	77 (77%)	102 (71.8%)	0.486
Non-ST-segment elevation myocardial infarction	63 (26%)	23 (23%)	40 (28.2%)	
Medical history				
Diabetes mellitus	66 (27.3%)	39 (39%)	27 (19%)	0.005
Hypertension	122 (50.4%)	45 (45%)	77 (54.2%)	0.171
Smoking	54 (22.3%)	15 (15%)	39 (27.5%)	0.086
Ischemic heart disease	38 (15.7%)	23 (23%)	15 (10.6%)	<0.001

Out of the participants, 142 (58.7%) showed no symptoms. The duration of symptoms was more commonly reported (43%) within a week before acute MI in the NSTEMI group and more than a month (32%) before in the STEMI group. Prodromal symptoms were mostly reported in the STEMI group compared to the NSTEMI group. Similarly, females exhibited more prodromal symptoms compared to males, and patients above 60 years of age reported more symptoms (see Tables 2, 3).

**Table 2:** Distribution of prodromal symptoms and the duration of symptoms by gender A P-value of <0.05 was considered statistically significant. Data presented as N (%). *Based on patients with prodromal symptoms

	Total (N)	Gender		P-value
		Male	Female	
Total (N)	242	174	68	-
Prodromal symptoms	100 (41.3%)	64 (36.8%)	36 (52.9%)	0.022
*Symptom type				
Chest pain (mild)	68 (68%)	47 (73.4%)	21 (58.3%)	0.120
Chest heaviness	44 (44%)	26 (40.6%)	18 (50%)	0.365
Chest burning	27 (27%)	16 (25%)	11 (30.6%)	0.548
Other types of pain	11 (11%)	7 (10.9%)	4 (11.1%)	0.979
Palpitation	42 (42%)	24 (37.5%)	18 (50%)	0.224
Unusual fatigue	23 (23%)	14 (21.9%)	9 (25%)	0.722
Sleep disturbance	22 (22%)	16 (25%)	6 (16.7%)	0.334
Shortness of breath	34 (34%)	23 (35.9%)	11 (30.6%)	0.586
Lightheadedness or dizziness	10 (10%)	5 (7.8%)	5 (13.9%)	0.331
Anxiety	23 (23%)	17 (26.6%)	6 (16.7%)	0.259
Sudden heat or flushing or a cold sweat	23 (23%)	15 (23.4%)	8 (22.2%)	0.890
Sudden back pain that is not related to an injury or exertion	22 (22%)	14 (21.9%)	8 (22.2%)	0.968
Sudden nausea or vomiting or unexplained indigestion	24 (24%)	18 (28.1%)	6 (16.7%)	0.198
*Duration of symptom				
1 week	32 (32%)	19 (29.7%)	13 (36.1%)	0.679
2 weeks	26 (26%)	16 (25%)	10 (27.8%)	
4 weeks	10 (10%)	8 (12.5%)	2 (5.6%)	
More than a month	32 (32%)	21 (32.8%)	11 (30.6%)	

**Table 3:** Distribution of prodromal symptoms and the duration of symptoms by age A P-value of <0.05 was considered statistically significant. Data presented as N (%) *Based on patients with prodromal symptoms

	Age			P-value
	<40 years	41-60 years	>60 years	
Total (N)	21	157	64	-
Prodromal symptoms	10 (47.6%)	59 (37.6%)	31 (48.4%)	0.274
*Symptom type				
Chest pain (mild)	7 (70%)	42 (71.2%)	19 (61.3%)	0.627
Chest heaviness	6 (60%)	21 (35.6%)	17 (54.8%)	0.122
Chest burning	3 (30%)	15 (25.4%)	9 (29%)	0.912
Other types of pain	2 (20%)	6 (10.2%)	3 (9.7%)	0.630
Palpitation	3 (30%)	23 (39%)	16 (51.6%)	0.370
Unusual fatigue	1 (10%)	9 (15.3%)	13 (41.9%)	0.010
Sleep disturbance	3 (30%)	16 (27.1%)	3 (9.7%)	0.134
Shortness of breath	3 (30%)	22 (37.3%)	9 (29%)	0.706
Lightheadedness or dizziness	0 (0%)	8 (13.6%)	2 (6.5%)	0.305
Anxiety	4 (40%)	13 (22%)	6 (19.4%)	0.388
Sudden heat or flushing or a cold sweat	2 (20%)	11 (18.6%)	10 (32.3%)	0.336
Sudden back pain that is not related to an injury or exertion	1 (10%)	10 (16.9%)	11 (35.5%)	0.082
Sudden nausea or vomiting or unexplained indigestion	1 (10%)	13 (22%)	10 (32.3%)	0.307
*Duration of symptom				
1 week	2 (20%)	18 (30.5%)	12 (38.7%)	0.650
2 weeks	4 (40%)	16 (27.1%)	6 (19.4%)	
4 weeks	2 (20%)	6 (10.2%)	2 (6.5%)	
More than a month	2 (20%)	19 (32.2%)	11 (35.5%)	

The most dominant symptom reported was chest pain, contributing to 68% of all symptoms, followed by chest heaviness, palpitations, shortness of breath, unusual fatigue, sleep disturbances, and anxiety. The association of prodromal symptoms with gender, age, and diagnosis type is presented in Tables 2-4.

**Table 4:** Distribution of prodromal symptoms and the duration of symptoms by diagnosis A P-value of <0.05 was considered statistically significant. Data presented as N (%) *Based on patients with prodromal symptoms STEMI, ST-segment elevation myocardial infarction; NSTEMI, non-ST-segment elevation myocardial infarction

	Diagnosis		P-value
	STEMI	NSTEMI	
Total (N)	179	63	-
Prodromal symptoms	77 (43%)	23 (36.5%)	0.367
*Symptom type			
Chest pain (mild)	52 (67.5%)	16 (69.6%)	0.854
Chest heaviness	35 (45.5%)	9 (39.1%)	0.592
Chest burning	22 (28.6%)	5 (21.7%)	0.517
Other types of pain	10 (13%)	1 (4.3%)	0.245
Palpitation	36 (46.8%)	6 (26.1%)	0.078
Unusual fatigue	20 (26%)	3 (13%)	0.196
Sleep disturbance	16 (20.8%)	6 (26.1%)	0.590
Shortness of breath	27 (35.1%)	7 (30.4%)	0.681
Lightheadedness or dizziness	8 (10.4%)	2 (8.7%)	0.812
Anxiety	17 (22.1%)	6 (26.1%)	0.688
Sudden heat or flushing or a cold sweat	19 (24.7%)	4 (17.4%)	0.466
Sudden back pain that is not related to an injury or exertion	19 (24.7%)	3 (13%)	0.237
Sudden nausea or vomiting or unexplained indigestion	19 (24.7%)	5 (21.7%)	0.772
*Duration of symptom			
1 week	22 (28.6%)	10 (43.5%)	0.541
2 weeks	22 (28.6%)	4 (17.4%)	
4 weeks	8 (10.4%)	2 (8.7%)	
More than a month	25 (32.5%)	7 (30.4%)	

## Discussion

In our study, the incidence of prodromal symptoms was 41% among the patients presenting with acute myocardial infarction, which is comparable to prior studies. Wood [5] reported a 45% incidence, and Solomon et al. [6] reported a 59% incidence.

The most dominant symptom reported was chest pain, accounting for 68% of all symptoms, followed by chest heaviness, palpitations, shortness of breath (SOB), anxiety, unusual fatigue, and sleep disturbance. The duration of symptoms was more common within a week before acute MI in the NSTEMI group and more than a month before in the STEMI group. In a study of 180 patients with proven myocardial infarction who were interviewed within 7-14 days of their admission about prodromal symptoms before myocardial infarction (they excluded patients over the age of 70), Stowers and Short found that 68% of them gave a history of unusual symptoms beginning two months before myocardial infarction. Of these, 55% reported chest discomfort or new-onset pain of a waxing and waning type, leading to hospital admission. Additionally, 13% reported other symptoms such as unexplained tiredness, breathlessness, intermittent claudication, palpitations, and ankle edema. The interval between the chest pain and infarction was between one and eight weeks [7].

Soltani et al. reported that 64% of patients experienced chest pain, 50% experienced unusual fatigue, and 20% experienced sleep disturbance [8]. In a comparative study of 337 participants, Heidarzadeh et al. reported 24 specific symptoms predicting acute coronary syndrome (ACS) in patients both with and without a history of ischemic heart disease (IHD) compared to healthy individuals. Some of these symptoms included chest pain or discomfort, palpitations, diaphoresis, vomiting, and SOB. Patients with a history of ischemic heart disease experienced 15 other symptoms more frequently, including SOB, discomfort in arms, orthopnea, and pain or discomfort in the jaw or teeth [9].

In our study, prodromal symptoms were mostly reported in STEMI patients. Solomon et al. reported that prodromata were more common in patients with non-transmural infarction and more common in females, and among STEMI patients, prodromal symptoms occurred more commonly with anterior or anterolateral wall MI patients (62% versus 38% in other locations) [6]. In a study comparing patients with acute anterior MI treated with primary percutaneous coronary intervention (PPCI), Ottani et al. found that those with prodromal symptoms had a limited infarct size and achieved thrombolysis in myocardial infarction-3 (TIMI-3) flow earlier than those without prodromal symptoms, suggesting that its presence leads to unstable thrombus or less activated platelets and ischemic preconditioning of the myocardium [10].

In our study, prodromal symptoms were most common in females, and common symptoms included chest pain, chest heaviness, and palpitations. McSweeney et al. studied 515 females diagnosed with acute myocardial infarction, aged between 29 and 97 years, and interviewed them four to six months after discharge to differentiate between prodromal and acute symptoms more effectively. Ninety-five percent of females experienced prodromal symptoms, and they experienced these symptoms more than one month before acute myocardial infarction. The most common symptoms were unusual fatigue (70%), sleep disturbance (47.8%), and SOB (42.1%). Only 29.7% of females reported chest discomfort and described it as aching, tightness, pressure, sharpness, burning, fullness, and tingling [11].

In one study, females waited longer than males to seek medical attention, presented with atypical symptoms, and were less likely to be admitted to the critical care unit (CCU)/ICU, resulting in higher mortality of female patients in the hospital [12].

Regarding risk factors, our study reported that prodromal symptoms were common in diabetics and in the female gender but were not associated with risk factors such as hypertension and smoking. This is consistent with a study by Soltani et al., where prodromal symptoms were not significantly associated with risk factors such as diabetes and hypertension but were strongly associated with the female gender [8]. However, some studies suggested that prodromal symptoms were associated with diabetes mellitus, family history of ischemic heart disease, and body mass index (BMI) [11,13].

In our study, prodromal symptoms were common in the elderly population because they may experience decreased sensory perception due to aging, polypharmacy, and the presence of multiple risk factors. This may explain the higher incidence of prodromal symptoms in this population, ultimately leading to delays in seeking medical attention before reaching the hospital [14].

In a case report of a 65-year-old diabetic female during her routine follow-up visit, she presented with a one-month history of recurrent nausea, vomiting, intermittent chest discomfort, and three weeks of insomnia. During this period, she visited the emergency department twice. Her differential diagnosis included gastroesophageal reflux disease (GERD), cholecystitis, and diabetic gastroparesis. Her abdominal ultrasound was unremarkable, but considering her risk factors and strong family history of ischemic heart disease, an echocardiogram and exercise tolerance test (ETT) were advised. The echocardiogram was unremarkable for ischemia, but the ETT showed ischemia. Her angiography revealed 99% stenosis in the left anterior descending (LAD) artery and 70% stenosis in the proximal right coronary artery (RCA). Emergent angioplasty was performed, thus protecting her from a fatal myocardial infarction [15].

Certain limitations of the study need to be acknowledged; it being a single center study with a relatively small sample size may limit the generalizability of study findings. Also, the relatively small sample size used in the study might affect the statistical power and precision of the results. Additionally, the study's cross-sectional design may restrict the ability to establish causal relationships between variables. Addressing these limitations in future research could enhance the strength and reliability of the study's findings and increase confidence in the generalizability and validity of the results.

## Conclusions

The findings from this study revealed that prodromal symptoms were present in a significant proportion of acute MI cases, with more than four in 10 patients reporting these early warning signs. Among the most frequently reported prodromal symptoms were chest pain, chest heaviness, palpitations, shortness of breath, unusual fatigue, and sleep disturbance. Importantly, these symptoms were observed to occur mostly within a week to over a month before the actual acute MI event. Early detection and prompt intervention based on these warning signs can play a crucial role in preventing acute MI episodes and saving lives.
